# Going for gold: Sports and exercise groups for people with dementia and carers contribute to their well-being

**DOI:** 10.3389/fresc.2022.953822

**Published:** 2022-09-01

**Authors:** Claire Chadwick, Aisha Hussain, Laura Carone, Jen Yates, Tom Dening

**Affiliations:** ^1^ School of Medicine, University of Nottingham, Nottingham, United Kingdom; ^2^ West Midlands Deanery, Birmingham, United Kingdom; ^3^ Mental Health & Clinical Neurosciences, School of Medicine, University of Nottingham, Nottingham, United Kingdom

**Keywords:** dementia, exercise, sport, psychosocial, quality of life, well-being, carers

## Abstract

**Background:**

Interventions involving exercise appear to have positive effects, both for people with dementia and for their carers. Quality of life and well-being are especially important outcomes. This study investigated how a sports and exercise group for people with dementia and their carers could contribute to the well-being of those attending the group.

**Methods:**

The study was a qualitative investigation, comprising semi-structured interviews, a focus group and observations. Participants included people with dementia and carers attending the group sessions, as well as staff providing the programme. The group sessions were provided weekly by the charitable trust of a leading sports venue. Data were analysed using thematic analysis.

**Results:**

A total of 16 participants were interviewed, including four people with dementia, eight carers, and four members of staff. Five main themes were identified: “Support to keep active and engaged is highly valued by people with dementia and carers”; “The challenges of being a carer are significant but sharing the experience really helps”; “People with dementia can have flourishing social lives”; “The group helps to maintain identity despite physical and role changes”; and “There are practical aspects of the group that make it appealing”.

**Discussion:**

People with dementia enjoy physical activity and experience the benefits of it. The sports and exercise group had an important role in providing access to activities that people with dementia and their carers value and enjoy, but would be difficult to undertake without a facilitated and safe environment. The group benefited the well-being of both people with dementia and carers in various ways, with peer support being of particular importance for carers.

## Introduction

Interventions involving exercise appear to have positive effects, both for people with dementia and for their carers. Quality of life and well-being are especially important outcomes. Interventions that provide opportunities for exercise and social interaction may positively affect well-being and alleviate behavioural and psychological symptoms (1). Exercise appears to benefit global cognitive functioning in people with Alzheimer's disease (2), and may improve performance of hippocampal related tasks (3) and activities of daily living in people with dementia (4). Since the gradual loss of function and behavioral symptoms accompanying dementia can significantly reduce quality of life (QoL) and well-being, psychosocial interventions that offer exercise in a group setting could be a useful way of maintaining or improving well-being.

There is evidence of a close and reciprocal relationship between the well-being of people with dementia and their carers' well-being and QoL (5, 6). Carers for people with dementia can have significant physical and psychological morbidity (7, 8); they have a high risk of stress (9), depression (10). A lack of time may lead to carers neglecting their own health (11) and becoming socially isolated. Carers often experience a lack of support (12) or when support is available, there are difficulties in accessing it (13). Dementia cafes, which promote social inclusion and support networks through interactions between carers, can promote increased social and mental well-being (14, 15). Other types of psychosocial interventions, such as exercise groups for people with dementia which involve carers, as either through active participation or by providing an opportunity to socialize with other carers, may be similarly beneficial.

Well-being is defined as a state of equilibrium that can be affected by adversities from life events but results in positive emotions and good mental health when achieved (16). It broadly encompasses physical, social, and psychological domains and consists of a variety of complex factors (17). Specifically, psychological well-being has been widely researched and according to “Ryff's six-factor model of well-being” it includes autonomy, environmental mastery, individual growth, positive relationships, life purpose and self-acceptance (18). This study aimed to explore how a sport and exercise group for people with dementia and carers (The Trent Bridge Community Trust project: “Forget Me Notts”) contributed towards their well-being.

## Methods

### Design and ethics

A qualitative design comprising semi-structured interviews, a focus group, and observations was employed to explore the perceptions and views of people with dementia and carers attending the group and members of staff or volunteers who facilitated the group. The sport and exercise group involved in the present study was the Forget Me Notts project, supported by the Trent Bridge Community Trust. This group enables people with dementia and carers to engage in various forms of physical activity in a safe setting. The sessions rotate between four venues: a cricket club, a leisure centre, a park with a pavilion, and a golf club. A variety of sports and activities are available including cricket, golf, badminton, tennis, football, seated aerobic exercise with music, and also quizzes and board games. The sessions last for one hour and generally take place twice a week, rotating between the different venues. During the study, the number of venues had increased from three to four, increasing the number of sessions provided towards the end of data collection.

A pragmatic position was adopted in this study, where the research team approached data collection, analysis, and interpretation with the perspective that the methods used were likely to result in practical ideas around what participants found positive, beneficial, helpful, or indeed the opposite, and that these ideas could be related to well-being for people with dementia and carers. Whilst it is acknowledged that truth and reality can vary amongst or within individuals, identifying general patterns across the data that fit with existing ideas around how people experience community groups would be appropriate to address the aims of this study.

Ethical approval for the study was granted by the University of Nottingham Division of Psychiatry and Applied Psychology Ethics Committee (reference no: 2819). Participants provided informed written consent for participating in interviews and/or focus groups, and only participants who were able to consent were included in this aspect of data collection. Written informed consent for participants to take part in observational data collection was provided on a separate form, either by participants themselves, or by their carers with a consultee consent form for participants who were unable to complete the form.

### Participants and sampling

Attendees of the Forget Me Notts group included people with dementia, carers, and group facilitators. Participants of this study were selected using purposive sampling of group attendees available during the sessions over a two-month period. Anyone attending or facilitating the group who was able to speak in fluent English and provide written informed consent was eligible to participate in the interviews and focus group. People with dementia who were deemed by their carers and/or group facilitators not to have capacity to provide informed consent were not invited to take part in interviews but were able to participate in the observational data collection.

### Data collection

Three members of the research team visited the group sessions weekly over a six-week period prior to data collection to introduce and familiarize themselves with the group attendees (CC, AH, and LC). This induction period also enabled piloting and refinement of the observational data collection technique.

Observational data were collected overtly by two researchers (CC and AH) who took part in the activities and then made notes during breaks in the sessions or at the end of the sessions, away from the participants. This technique enabled the researchers to immerse themselves in the activities and experience them in the way that participants did in addition to providing context and a rich understanding of how it felt to be part of the group. During the pilot phase, the researchers attempted to observe individual group attendees and make notes whilst the activities took place but found that this interrupted the flow of sessions and reduced the ability to capture interactions between participants. The researchers captured data regarding verbal and non-verbal communication, providing insight into participants' emotions and attitudes towards the sessions.

Semi-structured interviews were conducted with people with dementia, carers, and the lead group facilitator, and a focus group was conducted with three group facilitators plus the lead group facilitator. Interviews and focus groups took place at three of the venues over a period of two months and were conducted by two researchers (CC and AH). The interview schedule included a pre-prepared topic guide, developed from existing literature and refined in an iterative process during data collection (see Appendix A). Interviews took place in quiet rooms at the venues, and participants could choose to be interviewed individually or with the person they cared for/the person who cared for them present. Of the participants in this study, one person with dementia preferred to be interviewed with their carer present. The interviews were audio recorded and uploaded to a secure digital space at the University of Nottingham. Data were transcribed with the aid of an automated transcription service and anonymised upon transcription. Audio recordings were deleted upon completion of the analysis.

### Data analysis

The interview and focus group data were analysed using thematic analysis (19, 20) (see Table 1), and the notes from the observations were used to refine the themes and support interpretation. Initial analysis was undertaken by two researchers (CC and AH) and themes were discussed and refined with the wider research team (LC, TD, and JY).

**Table 1:** Stages of thematic analysis [after Braun & Clarke (19, 20)] and their implementation during data analysis.

Braun & Clarke suggested stages	Implemented in this project
Familiarising yourself with the data	Data were transcribed with the assistance of the Automated Transcription Service, edited to remove transcription errors, anonymized, and read thoroughly several time to familiarize
Generating initial codes	Initial codes and ideas were noted in the margin of the transcripts, comprising small statements or single words that captured the essence and meaning of the narrative
Searching for themes	Codes and ideas that addressed similar concepts were clustered together into groups and the overall sense of each group was noted
Reviewing themes	Groups that contributed similar yet distinct ideas were reviewed and categorized as sub-themes that contributed to substantial themes. Groups which stood alone were considered to be themes without supporting sub-themes
Defining and naming themes	Theme names were developed based on the codes and ideas in each contributing group and were refined as the narrative for each sub-theme or theme was created
Producing the report	Codes and ideas were used to develop the narrative for each theme, with representative quotes identified to support the narrative

## Results

**Figure 1:**
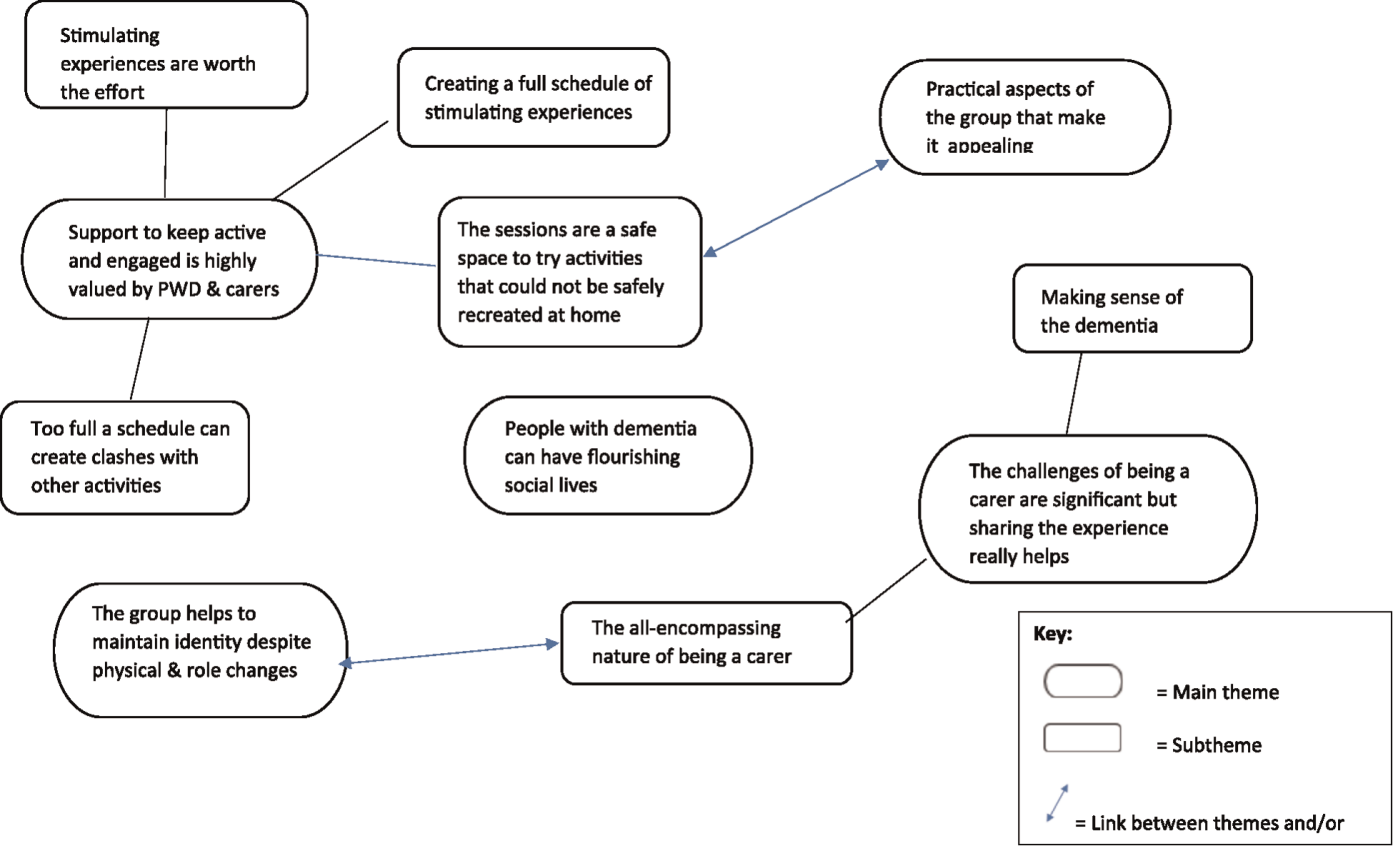
Theme map demonstrating the relationships between themes and subthemes.

Four people with dementia, eight carers, and the lead group facilitator were interviewed. The lead group facilitator plus three other group facilitators participated in the focus group. Participant characteristics are shown in Table 2. Five themes were identified during analysis which are shown in Figure 1.

**Table 2:** Participant characteristics.

Participant	Gender	Caregiver, Person with Dementia (PWD) or Staff	Relationship	Interviewed?	Observed?
1	Male	Carer	Sibling	Yes	Yes
2	Male	Carer	Spouse	Yes	Yes
3	Female	Carer	Spouse	Yes	Yes
4	Male	PWD	Spouse	Yes	Yes
5	Female	Carer	Spouse	Yes	Yes
6	Male	PWD	Spouse	Yes	Yes
7	Female	Carer	Spouse	Yes	Yes
8	Male	Staff	–	Yes	Yes
9	Male	Staff	–	Yes	Yes
10	Male	Staff	–	Yes	Yes
11	Male	Staff	–	Yes	Yes
12	Female	Carer	Spouse	Yes	Yes
13	Female	Carer	Spouse	Yes	Yes
14	Female	Carer	Spouse	Yes	Yes
15	Male	PWD	Sibling	Yes	Yes
16	Female	PWD	Spouse	Yes	Yes

### Theme 1: support to keep active and engaged is highly valued by people with dementia and carers

This first theme had four subthemes, as described.

#### Creating a full schedule of stimulating experiences

Participants were motivated to keep their diaries full up in order to avoid spending too much time sitting at home. Getting out and about and having a pattern of activities to their week was important to participants.

“*It's not good to be plonked in front of a television all the time.”(P12, Carer)*

*“Just getting out and having a pattern to work to and getting out on a regular basis, it's too easy to sit at home.”* *(P13, Carer)*

People with dementia valued the opportunity to keep physically active through the exercise opportunities provided by the group, and also enjoyed the mental stimulation through taking part in quizzes at the café sessions.

*“It [the Forget Me Notts group] keeps me physically active, it keeps my mind active, and I meet a lot of new people.”* *(P4, Person with dementia)*

For carers, there was a sense that it was important to enable their loved one to experience lots of different things, as though they were trying everything possible in the hope that they would land upon a particular activity that really engaged the person with dementia, and that as time is precious and potentially limited, there was a need to cram in as many things as they could before it was too late.

*“I wanted to try and do everything I could do that might possibly help, you know, prevent his deterioration … Anything I was offered I just said yes.”* *(P3, Carer)*

The tendency to prioritise was developed further as carers expressed that if they or the person they care for did not like a particular group, session, or activity, they simply didn't bother with it again, and instead saved their efforts just for the things that they liked to do most of all.

*“We thought we’d come, have a listen and see what it's all about and it we like it we’ll stay and if we don’t, we won’t.”* *(P2, Carer)*

The sessions provided three discrete points of enjoyment: firstly, the anticipation of going to the session, then the day itself, and lastly the impact after the session. Participants expressed that just having something to look forward to was helpful, even before having the positive and enjoyable experience at the sessions. One carer noted that having different things happening at the sessions each week was helpful, as this created anticipation and excitement, but also by not necessarily knowing the exact nature of the session, it reduced the opportunity for the person with dementia to talk themselves out of going. Once at the sessions, people with dementia and carers enjoyed the activities, and were able to have a laugh, experience moments of joy, and could live in the moment without thinking about dementia. There were lasting positive effects for people with dementia, as some had made memories and had new things to talk about with other family members or visitors.

*“He really enjoys it and when our son comes around in the evening, you know, he's full of what happened during the day, and he tells him about it”* *(P13, Carer)*

Even if a person with dementia couldn’t remember the session afterwards, carers still felt that being involved was positive, and in the absence of a cure for dementia, groups like this one made life feel a bit better. Carers gave a sense that their loved one would be tired after going to the sessions, but this was viewed positively, in a similar regard as to how we might feel after a fulfilling day.

*“After the sessions he's absolutely exhausted. It's brilliant, it's … it just gives him a new lease of life.”* *(P13, Carer)*

#### A schedule that is too full can create clashes with other activities

Whilst having a full schedule was viewed positively, this could create problems when people with dementia and carers were offered additional sessions or opportunities, as their diaries had little room for manoeuvre to fit other things in. Most people with dementia and carers were involved in other dementia specific groups (choirs, exercise classes, research studies), or due to having other co-morbidities, were involved in groups that supported people through different health conditions. Once participants knew where to find information about various groups, they found that there were many on offer, and they were able to fill up their time. However, they also acknowledged that time and the energy needed to get to groups is precious, leading them to prioritise the groups or sessions they felt benefitted them the most, or that offered them something different. Having more information about what would happen at particular groups or sessions was helpful in being able to plan and make the most of their time.

*“Sadly, we could only come once a fortnight because the memory café we go to. We like it there as well. I mean, it's totally different to this, much low key, but it's at the same time so we alternate”* *(P12, Carer)*

People with dementia and carers also had healthcare appointments to attend, which filled up their diaries further. Healthcare appointments were prioritised because participants had experienced waiting a long time for them, and consequently were reluctant to reschedule in order to get to additional sessions.

“*I have to take him to get his feet done, and I really don’t want to cancel that”(P14, Carer)*

Hobbies and interests contributed to a full schedule, where people with dementia maintained activities that were part of lives before their diagnoses. Carers kept independent interests or responsibilities that enabled them to have a break and engage in something meaningful to them that didn't involve caring. These activities were important, and often took precedence over attending groups.

*“So when [lead group facilitator] asked if we wanted to do this session on a Wednesday afternoon I said no I don’t think I can fit it in. That's usually my yoga afternoon”* *(P3, Carer)*

#### Stimulating experiences are worth the effort

Carers in particular reflected that getting to groups involved a considerable amount of effort. Sometimes this was due to practical aspects of the group itself, for example some venues were considered to be complex to access and this was off-putting for some carers. For most, the effort was related to the care required to help the person with dementia get up and ready for the day, and travel to the group. However, this effort was considered worthwhile in order to get to the groups. Carers and group facilitators reflected that people would travel longer distances and go to a lot of effort for good quality groups or sessions, or for groups that offered something new and different to what was being provided by other groups that people already attended.

*“This was the furthest that we had to travel … it didn’t affect because I would have travelled further to get [person with dementia] to a place that he would enjoy.”* *(P7, Carer)*

Participants attending this group in particular really valued the opportunities to exercise and this motivated them to make the effort as other groups they attended did not offer the same or similar levels of physical activity. Venues that were meaningful to group attendees, either because they were a local landmark, or because they had existing links with attendees, helped carers overcome the effort needed to get the person with dementia to the sessions. It felt good to go to these places, perhaps due to feeling part of the wider community, or being able to age in place. Some venues had additional activities taking place after the sessions, for example at the cricket ground group attendees were welcome to stay and watch any cricket taking place, which increased the value of going to the session and made the effort of getting there even more worthwhile.

*“Sometimes the cricket's on. Occasionally there is and in cases they’ve given us, let us stay you know when the sessions finish if cricket's on and he’ll let us go and sit in the stand for an hour say, and then we’ll probably go.”* *(P1, Carer)*

Carers and people with dementia thoroughly appreciated that the group exists, and expressed a sense of surprise that people would give up their time to run sessions for their benefit. They felt touched that people noticed their needs and helped them, and this motivated them continue attending.

*“I’m in admiration to somebody for setting it up and erm, because if you’re not actually living with dementia it's quite a something to do to set something up that you’ve not got anything to do with really.”* *(P5, Carer)*

#### The sessions are a safe space to try activities that could not be safely recreated at home

Most sessions run by this particular group involve a sporting or physical activity element, and this was valued by carers and people with dementia, who reflected that this was not something that they could achieve at home without the support of the facilitators.

“*The physical exercise I think. We wouldn’t do it if we didn’t come here”(P1, Carer)*

The activities could be tailored to different abilities and mobility levels so that everyone could feel that they were benefitting from it and this created a very inclusive atmosphere. Carers reported that people with dementia tended not to exercise at home, and that it could be hard to motivate them. Changes to people with dementia's concentration levels or their physical abilities that were needed to engage with hobbies and interests they had previously enjoyed meant that carers were often trying to find things for their loved ones to do to keep occupied, but they tended to have concerns about safety and which could result in them being overprotective. The sessions offered a safe space to do activities without the risk, which could take the burden away from the carers, and provided people with dementia and carers the opportunity to be themselves, and able to let go without the worry.

*“They allow people to do what they want to do, you know, without taking any risks”* *(P13, Carer)*

*“It gives the carers a chance to get out and not be sat at home and trying to think of things to do”* *(P3, Carer)*

Observational field notes obtained during the seated exercises supports this. Participants were concentrating as they were eager to get the actions right. There were lots of smiles and laughter throughout the activity especially when the task was difficult.

The group setting created an atmosphere where people with dementia were encouraged to try things, and often found that they could do more than they had believed. This helped people with dementia to develop their self-esteem and gain a sense of independence from their carers, particularly if they completed activities without their carers, even though carers were welcome to join in and often tended to.

*“It's given him a little bit of self-esteem, I think. In that he can do something on his own without me and that's why I haven’t taken part today. I’ve just let him go on and do it himself.”* *(P13, Carer)*

Some people with dementia had not developed sporting interests or hobbies throughout their adult life, and the sessions offered a chance to reconnect with activities from childhood that they might not have thought to do, or had the facilities to do, on their own at home. In order to develop the safe and inclusive atmosphere it was important that the physical activities and sports were those considered “softer”, such as table tennis, badminton, golf, and gentle football. Carers and group facilitators reflected that a different group that had run previously was too strenuous, and didn't feel safe, demonstrating the importance of finding the right balance of physicality.

*“We quickly sort of learned that er where they were always with different mobility issues and spatial issues that they weren’t working and I seemed to spend more time at [the local hospital] where they bumped heads than actually running sessions, so we decided to … [do] softer sports”* *(P8, lead group facilitator)*

One person with dementia considered that group attendees might feel less confident to reach out and suggest ideas for activities so it was important that facilitators provided a variety of activities to cater for different interests and abilities.

The group offered a choice of activities to the attendees. Observational data collected from one session described participants being given the choice between taking part in seated exercises and using indoor cricket equipment. Participants with greater mobility chose to use the cricket equipment whilst those who were less mobile chose the seated exercises.

The physical activities did not necessarily have to be sports. Participants were observed throwing dodgeballs at a giant gym ball with the aim being to get the gym ball across the opposing team's line. If successful, the team obtained a point.

Carers and people with dementia valued the opportunity to learn new skills but highlighted the need to show people what to do rather than assume that they would know, as they might not have done it before or for a long time.

*“You pick it up, but you don’t play it well because you don’t know how to play it but then people have showed me when I’ve done things wrong how to play it”* *(P1, Carer)*

### Theme 2: the challenges of being a carer are significant but sharing the experience really helps

This theme had two subthemes, one concerning the nature of being a carer and the other about making sense of dementia with the support of other carers.

#### The all-encompassing nature of being a carer

All carers interviewed in this study spoke of the challenges of being a carer. This was echoed by the group facilitators, and also by people with dementia, who were very aware of the pressure that their dementia placed on their loved ones.

*“I drive my wife mad because she’ll tell me something and I acknowledge it and ten seconds later I’ll say “What did you say?”. That's the problem.”* *(P4, Person with dementia)*

Key difficulties of caring were being able to find out information and access support, where carers felt that they struggled to find what they needed to know unless they had a family member helping them, and that this lack of information could create problems for them later on as their loved one's dementia progressed or after they had died. Sometimes, the reverse was also true, where carers were bombarded with a large amount of information and the number of groups available suggested a crowded marketplace which made it difficult to prioritise what sessions to attend.

*“When he was first diagnosed, I was bombarded with information and I couldn’t, you know, I don’t know what came from where.”* *(P3, Carer)*

The caring role had gradually built up for most carers and the person they cared for had lost independence over time, with their needs intensifying. For some carers who were not spousal partners, this involved the person with dementia moving into their homes. For other carers, their role had increased to helping with personal care, making sure their loved one ate, and managing continence issues.

*“I have to help him shower, dress. He wouldn’t take his medication if I didn’t give it him. He wouldn’t eat, wouldn’t drink.”* *(P5, Carer)*

Carers found it harder to encourage their loved ones to do activities, and in some instances the person with dementia could get frustrated or miserable because it seemed like the carer was always telling them what to do. This contributed to feelings of guilt and frustration for the carers, accompanied by feeling very tired of constantly needing to be aware of what their loved one was doing. The group, however, offered a change of dynamics, and this was a welcome relief to carers.

*“He was quite anti me, which is quite common because I’m the one who's saying here's your food, let's have a shower, let's do that, and they don’t want it … he could be quite a misery at home but as soon as we got out, or as soon as somebody else came in, it was just a change of scenery from it, a change of the dynamics.”* *(P7, Carer)*

Having the facilitators take on the role of engaging the people with dementia in activities took away some of guilt, and some carers felt comfortable to leave their loved ones at the group whilst they had time to themselves for their own well-being. The group also offered information sessions for carers, and the facilitators were on hand to provide support or find out further information if carers needed it.

#### Making sense of the dementia journey with other carers

The group that our participants attended enabled carers to get together whilst their loved ones were engaged in other activities and share their own stories, experiences, and advice, and this was considered to be a very positive aspect of the group. Carers considered that whilst their loved ones might be at different stages of dementia, they were all in a similar boat and had a lot to offer each other.

*“I’ve taken part in what's been going on as well, and I’ve met other wives with the same, similar problem to me, and it just makes you realise you’re not on your own, but there are other people out there and it's just nice to talk to somebody and find out how they’re coping.”* *(P13, Carer)*

For some, this was enacted through a positive comparison where carers considered that their loved one was not experiencing significant changes and could manage well in comparison to other people with dementia at the group. Others considered that this was a glimpse into the future, where they could see what might be ahead of them and prepare for it. One carer reflected that as he spent so much time with his wife he was less sensitive to the changes that could happen with her dementia, and the other carers helped him to be aware of what to look out for.

*“Sometimes I don’t notice any difference from the day she was diagnosed … when you’re with somebody every day, it's just occasionally you notice things”* *(P2, Carer)*

Some of the female carers made sense of this through remembering what it had been like to be a mum when their children were younger, finding it to be a congruent experience with swapping stories with other mums.

*“Yes, and meeting the other carers you know. Swapping stories with them, it's like when the children were babies, you know the toddler group. You got together with other mums and you discussed things, and things like that.”* *(P3, Carer)*

### Theme 3: people with dementia can have flourishing social lives

The social aspect of attending the group was important to people with dementia and carers, and it was recognised by the group facilitators too.

*“Believe it or not, they make friends as well … it's great to see people with dementia making friends with other people as well with dementia. They’re not just closed off, doing their own thing.”* *(P9, Group facilitator)*

The group attendees bonded with each other and with the group facilitators to become friends, and a key ingredient in the success of this was the non-stigmatising approach taken by the group towards dementia. People with dementia were not treated differently, with attendees commenting that people wouldn't know that people have dementia as no-one stands out because of it.

*“A lot of people, you wouldn’t know they’ve got dementia. And unless you see their movements or the way they react, nobody ever stands out because of it.”* *(P2, Carer)*

The ability to meet other people was important to people with dementia, and carers felt it was important for their loved ones to have these opportunities, particularly following the pandemic where social interactions were limited. Participants reflected that whilst they might not have chosen to come to this group if they didn't have dementia, it gave them the chance to meet new people who were in a similar situation and develop a shared understanding, so in a way having dementia had opened doors to new friends.

*“I meet other people I can understand … it makes me recognise that there are other people that are also in the same boat, or in some cases worse than me, some cases better than me. But I learn from other things as well.”* *(P4, Person with dementia)*

Meeting new people would be difficult to do without the group as people with dementia could often spend quite a lot of time on their own or at home.

*[When asked about their main reason for coming]: “Well to interact basically. Because I’m on my own all the time. It's a chance to interact with people and talk to them.”* *(P15, Person with dementia)*

Furthermore, field notes from observational data highlighted how sporting activities such as indoor cricket and other team ball games involved all the attending participants. The collaborative nature of these sports seemed to facilitate an opportunity for participants to socialise with each other.

Taking part in the activities at the group was considered to be a helpful approach to socialising. It could feel daunting to initiate a conversation with another group attendee but it was easier to chat whilst engaged in an activity. This was especially the case when talking to new group attendees that people with dementia had not met before or did not know well, as the activities pushed them to join in rather than gravitating towards the faces they already recognised. One participant reflected that being in a group and being able to contribute to the conversation was helpful, perhaps if their confidence was low because it could take the pressure away from having to begin the conversation.

*“It takes me a while to sort of start talking to people. If I’m in a group of people, I do talk, but mostly I listen.”* *(P16, Person with dementia)*

A variety of different activities on offer at the different sessions encouraged different people to attend which increased the opportunities to meet new friends, although having continuity of group attendees was also considered helpful to increase confidence when making connections. Participants found it reassuring to see faces they knew, even if they had forgotten other group attendees' names.

“*I know people's faces. I can’t remember the names. It's just reassuring.”(P15, Person with dementia)*

Having plenty of group attendees was thought to be necessary for creating the social atmosphere at the group, as when the numbers of people attending the group were small the feel of the group could be a bit flat. Participants reflected that it was sad when some people had stopped coming to the sessions upon moving into residential care, as they had bonded with them and wouldn't get to see them again. During the pandemic the group attendees had missed being able to see each other, and after the sessions had resumed some group attendees were no longer there. Participants felt it was important to know how others were, and were disconcerted when they were replaced by new faces.

*“You lose touch with people. You don’t know how they’ve been doing or how things have been happening with them. And so when you come back you suddenly find that there are new faces. The old faces, have not, not here.”* *(P16, Person with dementia)*

### Theme 4: the group helps to maintain identity despite physical and role changes

People with dementia and carers experience changes to their physical abilities associated with ageing, or with the progression of their dementia, and they can also find that their social roles change as the expectations and responsibilities upon them alter. The group, and the activities on offer, provided opportunities to reconnect and maintain aspects of their identity. For example, some people with the dementia considered themselves, or were considered by their carers, to be outgoing and sociable people, and the group enabled them to enact this part of their identity through the social aspects. People with dementia could still view themselves as someone who liked to meet others and the group offered a way of achieving this that might be different to how they had approached it earlier in their lives, but still leading to the same outcome.

“*I can meet a lot of people. I’m a very outgoing person, I talk to anybody.”(P4, Person with dementia)* *(P4, Person with dementia)*

*“You’ve probably noticed, he's a very sociable animal, and it's that aspect that I think he likes”* *(P3, Carer)*

Many people with dementia attending the group had particularly enjoyed sport, either throughout their whole lives, or during their childhood. The activities on offer at this group enabled them to reconnect with this part of themselves. Some people with dementia considered themselves to be active people, and being able to safely participate in physical activity was congruent with how they saw themselves.

“*I’ve always been an active person, so I still enjoy doing things”(P4, Person with dementia)*

*“He's always loved sport, so he used to play some pro football, he used to love rugby at school. Er, has played squash. Used to run half marathons.”* *(P5, Carer)*

Observational field notes demonstrated the vast choice of sports and activities available to participants. At one venue participants were either playing darts, badminton, tennis, table tennis and football with a member of staff. Other participants were playing Jenga or chatting with others.

Identity was also realised through the collaborative nature of the group. For example, one participant had enjoyed ownership of a business earlier in their life, and being able to put forward ideas and have them implemented could help to retain this sense and view of the self.

“*I feel that if I want them, I suggest something in the groups and they’re taken on”(P4, Person with dementia)*

Identity was important for carers, who reflected that being a carer could take up someone's whole identity and they risked losing their own sense of self because they couldn't engage with their own hobbies. Being able to leave their loved ones at the group created time for them to be involved in their own interests.

*“The carers can actually be themselves for what, an hour … The carers actually trust us with their partners.”* *(P9, group facilitator)*

Group facilitators also reflected on the identity and sense of self of the people attending the group by looking at what skills, interests, and ideas people with dementia had there and then, instead of thinking about what they had lost as their dementia progressed. The facilitators recognised that people with dementia were more than just their diagnoses and enjoyed finding out about their lives.

“*They’ve lived a life. It's really good to listen to, their stories and what they’ve done.”* *(P8, Lead group facilitator)*

*“There's some really amazing people here when you think about their lives.”* *(P10, Group facilitator)*

This was echoed by carers, who appreciated the reminiscence sessions because they provided an important opportunity to remember who their loved ones were before they were diagnosed with dementia.

*“You could easily forget you know. You have to think back to before they had this problem and think of those memories. So it is important that you keep those going, photographs and things.”* *(P12, Carer)*

### Theme 5: there are practical aspects of the group that make it appealing

Various aspects of how this group was organised and run made it appealing to both people with dementia and carers. As mentioned previously, the non-stigmatising nature of the group created a welcoming feeling that took the focus away from dementia and instead helped attendees to focus on what they could do. Participants commented that their interactions with group facilitators never felt forced, and that group facilitators didn't hold back from interacting with anyone and didn't draw attention to anyone's dementia. The overall feeling of the group was one of kindness, where group facilitators and group attendees were genuine, friendly, and approachable, and group attendees felt as though they were being looked after. This feeling could be due to the group facilitators getting to know everyone that attending, and taking time to welcome any new group attendees when they first arrived.

“*We’re all welcoming so as soon as someone comes in, someone is there to greet them”(P9, group facilitator)*

*“As soon as we pulled up [the lead group facilitator] was on the front to meet us and I thought that's really kind.”* *(P12, Carer)*

Practical aspects were helpful, such as having convenient locations to run the sessions, which generally had good parking facilities or were accessible by public transport. Participants commented that they liked having refreshments provided, and that the availability of these helped group attendees to socialise because it was easier to talk over a cup of tea. Some group attendees had found out about the group from attending other dementia specific groups, and noted that whilst this group had specific activities that were not provided by other groups, after a while all the groups did blend into one but in a helpful and complementary way. Sometimes a trusted member of the wider dementia community would recommend something, such as this group, and that gave group attendees confidence that this would be good quality and worthwhile attending.

## Discussion

Overall, the themes identified in these data suggest that groups have an important role in helping people with dementia and their carers participate in activities that they value and enjoy but would find difficult to do at home without support from group facilitators. Groups such as Forget Me Notts provide carers with an opportunity for social interaction and to receive much needed support (21) which increases social and mental well-being for carers (14). The opportunities for people with dementia to fulfil their social needs, for both people with dementia and their carers to re-establish their identity, maintaining their own sense of self in addition to re-establishing their relationship occurs alongside participating in the physical activities. This supports the finding of Camic, Tischler & Pearman (22) who conducted a study into the experiences of an art-gallery intervention for people with dementia and their carers, which concluded that this enhanced the relationship between the PWD and their carer. Whilst the sporting activities on offer in this group might be the initial motivation to attract group attendees, it is the overall package of beneficial physical activity plus social and emotional support that creates a positive impact on well-being.

Physical exercise can be linked to a variety of biological and psychological mechanisms which improve well-being and QoL in individuals of any age (23). People with dementia may be unable to routinely access these benefits due to difficulties in undertaking exercise. These may be due to cognitive or physical changes, where recent research suggests that apathy can be a significant barrier to engaging with activities and interests (24), or due to fear of embarrassment or a lack of support (25). The group benefited the well-being of people with dementia by creating the opportunity to do physical activity and highlighted that with appropriate adaptations of activities and a strong support network, engagement in activities of people with dementia is much greater. This aligns with Ryff's six factor model of well-being by demonstrating the factors of environmental mastery and autonomy (18). The group further impacted on the well-being of carers by taking the pressure away from them in having to motivate their loved one to do particular tasks.

Furthermore, increased participation in hobbies is associated with better well-being for a person with dementia (26), and attending this group enabled participants to reconnect with sporting interests that they had enjoyed across their lifespan. Familiar preferences and practices like hobbies are said to make up aspects of one's selfhood (27), and having a strong sense of self is important in preserving well-being (28). This may fit with Ryff's model through demonstrating life purpose and also individual growth in situations where people with dementia have developed skills (18). Although more evidence for this link is required, losing interests and pastimes may lead to a reduction in the well-being of a person with dementia, so the opportunity to revisit them could be considered to maintain or improve well-being.

The link between loneliness and dementia is complex, with inconsistent findings (29). Previous literature suggests that whilst developing interpersonal relationships through social engagement is important in overcoming loneliness, putting people with dementia in unfamiliar environments with new people could be unhelpful (30). However, the findings in this study suggest that this group was able to mitigate such challenges through the inclusive nature of the sessions, allowing carers to attend alongside the person with dementia, and using activities to create opportunities to socialise, which may have made the environment feel more familiar. Although it is uncertain whether groups for people with dementia can reduce loneliness, our data suggest that participants felt less alone, and this could enhance their overall well-being (31), again linking to Ryff's model through the factor of positive relationships (18).

Due to the progressive nature of dementia, people with dementia generally require increasing levels of care over time. Caring is challenging and may result in stress and frustration, with several studies linking carer burden with a poorer QoL (32, 33), since caring impacts upon factors such as having time for oneself and maintaining social networks. The group was valuable to carers through creating time, as carers felt confident to leave their loved one whilst they did other things, and it was also a valuable social network that provided access to informal support from other carers. This is likely to have enhanced the well-being for carers, and in turn may impact positively on people with dementia.

### Strengths and limitations

A key strength of this research was the use of a qualitative design, which enabled a thorough exploration of participants' meanings. Thematic analysis allowed for a combined deductive and inductive approach, where analysis could be supported by existing literature and combined with new, data driven insights. The combination of the interview and focus group data with the observational field notes provided a deeper understanding of perceptions, and increased confidence and credibility of the findings. Initial pilot work enabled the researchers to develop a thorough understanding of how the groups operated and to build rapport with potential participants which may have increased their confidence and comfort when participating in data collection.

Purposive sampling was used to recruit participants who were available at the group, and does mean that potential participants who were unable to attend, or who had stopped attending the group, were not invited to take part in the study and therefore their views are not known. As participant recruitment and data collection took place over several sessions, it is likely that a good range of participants were offered the opportunity to participate, and further studies may seek to understand why some individuals discontinue attendance at groups. The sample has a greater number of carers than people with dementia and therefore it could be said that carers are more represented in this study than people with dementia. Whilst this could be seen as a limitation, an aspect of the project focused upon the carers' perspectives and it successfully demonstrates the differences in what carers and people with dementia value the most. Carers emphasised how the group provided them an opportunity to have a break whilst people with dementia valued being able to participate in activities. The sample included in this study were from one geographical area. The findings are therefore unable to indicate how people with dementia and carers from other areas or from different communities might experience groups providing sports and physical activities, and what their preferences for such activities are.

The themes and subthemes that are identified in this study do not specifically highlight the traditional benefits, practicalities and challenges of sport and exercise that are often considered outcomes in research regarding sporting interventions for people with dementia and their carers. Instead, these findings identify softer benefits of sport and exercise, and demonstrate that sport can be an important draw to increase attendance at social groups. Whilst a focus on social aspects in this study could be seen as a limitation, it demonstrates what people with dementia and carers value the most from attending the sessions and future work should seek to incorporate a wider range of outcomes when evaluating the success of sporting activities.

### Implications for research, theory, and practice

The findings suggest that both people with dementia and carers benefited from attending the group, and that it positively impacted their well-being in different ways. People with dementia can find it difficult to access physical activity at home or without support because it places pressure on carers to plan such activities and then carry them out safely. The opportunity to come to a group facilitated by trained professionals in a safe environment enables people with dementia to realise the benefits of engaging in physical activities and as such, more research is needed into whether similar groups can be supported in additional areas. For carers, the opportunity to receive support and a shared understanding from other carers was important, particularly within a context of difficulties in accessing formal support. An evaluation of the provision of support available for carers and its ease of access is required. Easier access to support for carers both whilst caring for the person with dementia and which aids in adjustment once the person with dementia has died is necessary such as through the implementation of similar groups. Further research could seek to explore whether informal support networks provided through groups mitigate the lack of formal support, and how informal support can operate to enable access to formal services. Easier access to support through informal channels could reduce barriers to well-being commonly reported by carers.

The social aspect of coming to the group was important to people with dementia despite the focus of the group being sports and physical activity. The participants in this study recognised their need to socialise and found the activities on offer to be a helpful vehicle that brought them together and created opportunities to interact. Many groups for people with dementia provide time and a space to talk with others, but it could be helpful to facilitate activities at the same time, in order to make social opportunities feel more natural and accessible for people who may be less confident.

This study highlighted the practicalities to be considered when developing groups for people with dementia and carers, such as location, public transport links, and start time of the session. It would be beneficial for existing dementia friendly groups to be evaluated to ensure these important factors are implemented in addition to implementing these factors when developing similar dementia friendly groups. This will encourage attendance which is shown to have positive social benefits for both people with dementia and carers. Whilst this study was undertaken in in one geographical area with participants who had similar demographics, it is likely these aspects can be applied to other communities for use in the development of similar dementia friendly groups, but further research may be needed.

## Conclusion

People with dementia enjoy physical activity and experience the benefits of it. Groups providing this kind of activity represent a stimulating and valuable experience that carers are prepared to go to considerable effort to access, because they recognise that without such opportunities the person they care for would struggle to participate in physical activity or exercise. Alongside the physical benefits of participating, the activities create social interactions for people with dementia, and enable carers to access informal support and create a shared understanding of their dementia journey.

## Data Availability

The raw data supporting the conclusions of this article will be made available by the authors, without undue reservation.

## Appendix A: interview schedule

### Outline interview or focus group schedule

#### Brief introduction

First of all thank you very much for agreeing to be interviewed and helping with this research project. As the information sheet explained we want to ask you about the dementia friendly sports sessions you attend/help with. Before we start please can I have your permission to record this interview? The interview will be transcribed and any information which could identify you such as your name will be removed, the recording of the interview will then be erased.

If it is okay with you I will start by asking you some questions about yourself as it is useful to know about who you are, when we explore your opinions.

Topic guide for interviews with people with dementia, their carers, organisers/supporters of the dementia friendly sports sessions.

#### Topics for carers

Please can you tell me a bit about yourself?

(Prompt for information relating to relationship to pwd, how long have you been a carer, time spent caring, any help/respite received, other groups/sessions attended)?

Can you tell me a bit about the person you care for?

(Prompt for age, type of dementia, when diagnosed, occupation, past and present hobbies)

How did you find out about the sessions?

Why did you start attending?

What has been the impact of the group on you?

Impact of the group on participant with dementia? (Prompt for what do you notice before, during, after the sessions?)

Many carers of people with dementia feel lonely and isolated is this something you have experienced?

(Prompt for can you describe how this feels, have these sessions made any difference and if so how and in what way?)

Do you have any comments on the organisers supporters of the group?

(Prompt for how do they help you during the session, interact with pwd, enhance the experience?)

The sessions are termed “dementia friendly” how did you find them?

(Prompt for what if anything could the staff do to make it more dementia friendly? Any aspects of the venue or organisation of the sessions which could make them more dementia friendly?)

Did you ever come and watch cricket match here previously at Trent Bridge?

Did the fact the session were linked with Trent Bridge cricket club encourage you to attend? If so why?

Did the fact some of the sessions were held at Trent Bridge cricket club make you more likely to attend? If so why?

What do you think about the group name “”?

#### Topics for organisers/support staff

Please can you tell me a bit about yourself?

(Prompt for information relating to how long have you worked here, time spent on this project, any other specialist group/sessions been involved with?)

When did you first get involved with the sessions?

Why did you get involved?

(Prompt for part of the job, development opportunity, new opening, interest or experience of dementia.)

What do you know about dementia in terms of any personal experience?

What special training/information have you had on dementia to prepare you for these sessions?

What if any special training have you had about how to make the sessions dementia friendly?

What if any training have you had about how to make the facilities dementia friendly?

What if any extra training would you like/or feel you need to improve these sessions?

How do you feel about the sessions?

(Prompt for enjoy them, find them frustrating/depressing/inspiring/satisfying)

What works about the sessions and why do you think that is?

What doesn't work about the sessions and why do you think that is?

What impact do you think the sessions have on the pwd and their carers?

(Prompt for what makes you say that?)

How would you like to develop the sessions if time and money were not an issue?

Do you think having the sessions linked with Trent Bridge cricket club has encouraged people to come and if so why?

Do you think having some of the sessions at Trent Bridge cricket club makes people more likely to attend? If so why?

What do you think about the group name “”?

### Topic guide for participants with dementia*

Have you always been into sport (Prompt for did you participate in any sports clubs as a child, adult, do you follow any major sporting tournaments, if so what?)

Were you aware of Trent Bridge cricket club prior to attending the group?

Did you ever come and watch cricket match at Trent Bridge previously?

Why do you come to these groups?

(Prompt know the stadium, easy to get too, friends come)

What do you do during the sessions?

How do you feel about the sessions?

Do you like coming? Why?

Do you like the staff? Why?

What's good?

What's not so good?

Do you go to any other physical activity, or group activity?

How do these sessions compare to other groups or activities you go to?

Anything they could do to make the experience better?

What do you think about the group name ““

Other interests
